# High-frequency neural oscillations and visual processing deficits in schizophrenia

**DOI:** 10.3389/fpsyg.2013.00621

**Published:** 2013-10-09

**Authors:** Heng-Ru May Tan, Luiz Lana, Peter J. Uhlhaas

**Affiliations:** ^1^Institute of Neuroscience and Psychology, College of Science and Engineering and College of Medical, Veterinary and Life Sciences, University of GlasgowGlasgow, UK; ^2^Brain Institute, Federal University of Rio Grande do NorteNatal, Brazil; ^3^Department of Neurophysiology, Max-Planck Institute for Brain ResearchFrankfurt, Germany; ^4^Ernst Strüngmann Institute (ESI) for Neuroscience in Cooperation with Max Planck SocietyFrankfurt, Germany

**Keywords:** schizophrenia, high-frequency neural oscillations, visual perception, neurobiology, evoked and induced neural activity, neural synchrony

## Abstract

Visual information is fundamental to how we understand our environment, make predictions, and interact with others. Recent research has underscored the importance of visuo-perceptual dysfunctions for cognitive deficits and pathophysiological processes in schizophrenia. In the current paper, we review evidence for the relevance of high frequency (beta/gamma) oscillations towards visuo-perceptual dysfunctions in schizophrenia. In the first part of the paper, we examine the relationship between beta/gamma band oscillations and visual processing during normal brain functioning. We then summarize EEG/MEG-studies which demonstrate reduced amplitude and synchrony of high-frequency activity during visual stimulation in schizophrenia. In the final part of the paper, we identify neurobiological correlates as well as offer perspectives for future research to stimulate further inquiry into the role of high-frequency oscillations in visual processing impairments in the disorder.

## Dysfunctions in visual perception in schizophrenia

Disturbances in visual perception were for a long time considered relatively unimportant in the understanding of schizophrenia (ScZ) compared to the more striking clinical presentation of hallucinations and delusions. Bleuer summarized this view as follows: “Sensory responses to external stimulus are quite normal. To be sure, the patients will complain that everything appears to be different. However, this strangeness is usually attributable to a deficit in customary associations and particularly to an alteration of emotional emphasis.” (Bleuler, 1969; page 76). Similarly, Kraepelin (1919) concurred that “[P]erception of external impressions in dementia praecox is not usually lessened to any great extent as far as a superficial examination goes.” (Kraepelin, 1971; page 5).

Following evidence from phenomenological research which indicated profound alterations in perceptual experience (see Uhlhaas and Mishara, 2007 for a review), an increasing number of studies began to investigate sensory processing experimentally (Place and Gilmore, 1980). Since then, a large body of evidence has accumulated that has highlighted impaired visual processing as a core deficit in schizophrenia (Klosterkötter et al., 2001; Javitt, 2009). Such dysfunctions involve the discrimination of orientation, motion, and object size (e.g., Butler and Javitt, 2005; Butler et al., 2007, 2008; Chen, 2011), which have been related to the magnocellular pathway because of reduced sensitivity to stimuli with low spatial frequency (Schechter et al., 2003; Butler and Javitt, 2005).

Moreover, ScZ-patients show reduced contextual influences in relationship to contrast (Yang et al., 2013), motion (Tadin et al., 2006), orientation (Yoon et al., 2010) as well as during contour-integration (Uhlhaas et al., 2006b), which could underlie impairments in perceptual organization (Uhlhaas and Silverstein, 2005). Additional visual processing deficits in ScZ have been revealed by masking paradigms (Green et al., 2011) which have highlighted longer intervals between the target and mask stimuli for accurate identification of targets (e.g., Green et al., 1994, 1999). Importantly, visual dysfunctions have been linked to impairments in higher cognitive functions (Javitt, 2009), such as working memory (Haenschel et al., 2009). Moreover, there is evidence to suggest that changes in visual perception are related to more complex features of the disorder, such as the development of delusions and changes in self-experiences (Uhlhaas and Mishara, 2007).

Data on abnormal visual functions in ScZ is consistent with evidence on anatomical abnormalities as revealed by post-mortem studies (Selemon et al., 1995; Selemon and Goldman-Rakic, 1999; Dorph-Petersen et al., 2007) as well as magnetic resonance (MR) and Diffusion Tensor Imaging (DTI) studies (Staal et al., 2000; Clasen et al., 2003; Arnone et al., 2009; White et al., 2011; Whitford et al., 2011b). These findings suggest that in addition to abnormalities in fronto-temporal regions, alterations in anatomical parameters extend to early visual areas. More recently, electro/magnetoencephalography (EEG/MEG) and functional magnetic resonance imaging (fMRI) have disclosed corresponding deficits in neural responses during visual stimulation (Spencer et al., 2003; Wynn et al., 2005; Uhlhaas and Singer, 2006; Uhlhaas et al., 2006a,b; Yeap et al., 2006). Specifically, studies assessing event-related potentials (ERPs) have demonstrated impairments during early and later visual processing stages in ScZ (Box 1).

Box 1 **Event-related potentials (ERPs), visual perception, and Schizophrenia**

*ERP* waveforms are commonly derived in EEG studies that investigate the neurophysiological mechanisms underlying sensory perception. ERP waveforms consist of a series of transient positive and negative voltage deflections that are time-locked to stimulus onset. These transient fluctuations in ERP waveform polarity are conventionally extracted as basic components and named by their polarity in conjunction with either their latency or ordinal position, relative to stimulus onset. In general, early components (e.g., those occurring before ~200 ms) are thought to reflect early sensory processing while higher cognitive processes are related to later components.

*N80*—an initial negative deflection that peaks ~70–90 ms post stimulus onset (Di Russo et al., 2007)—is considered the earliest visual ERP component and thought to be mainly driven by parvocellular (P) input but likely with small influence from the magnocellular (M) pathway in response to visual stimuli consisting of higher contrast levels (Foxe et al., 2008). In a recent investigation, using a range of visual stimuli that theoretically bias M or/and P pathways using systematic manipulation of stimulus luminance, chromatic contrasts and flicker, Núñez et al. (2013) demonstrated that the occipital N80 component in early-onset (but not adult-onset) ScZ was significantly lower in amplitude in response to stimuli that involve both M and P pathways. They also observed significantly prolonged onset of N80 latency in adult-onset ScZ. In contrast, the N80 amplitude in response to isolated P- and M-biased stimuli was comparable in both healthy controls and ScZ patients. The finding is suggestive of a deficit in M-priming on the P pathway in early-onset ScZ.

*P1*—a component with positive deflection ~100 ms post visual stimulus onset—is known to involve the dorsal and ventral visual streams (Martinez et al., 1999; Di Russo et al., 2002). In response to visual stimuli (e.g., motion, spatial, low contrast) that bias the magnocellular pathway, the occipital P1 amplitude is commonly reported to be markedly decreased in patients with ScZ (e.g., Doniger et al., 2002; Schechter et al., 2005; Butler et al., 2007; Lalor et al., 2012; Núñez et al., 2013; however, see e.g., Johnson et al., 2005; Wynn et al., 2008, also mentioned below). Prominent P1 amplitude reduction was also observed in ScZ patients when they engaged in visual tasks involving illusory contour processing (Foxe et al., 2005) or fragmented object recognition (Doniger et al., 2002), and this reduction paralleled the weaker scalp activations over their lateral and posterior occipital areas.

*N1*—a negative component peak that manifests ~150–200 ms—is thought to predominantly reflect ventral stream processing (e.g., Doniger et al., 2002). Many studies that have assessed the N1 component, e.g., using illusory contour or fragmented contoured stimuli for object recognition, have reported comparable N1 amplitudes between ScZ patients and controls (e.g., Foxe et al., 2001; Doniger et al., 2002; Foxe et al., 2005), suggesting that parvocellular-mediated ventral stream processing is largely unaffected. However, studies that investigated face processing in ScZ patients have demonstrated pronounced reduction in their N170 amplitude in response to face vs. building stimuli (Herrmann et al., 2004b; Turetsky et al., 2007). Similar prominent reduction in N150 amplitude was observed in ScZ patients engaged in local vs. global visual perceptual tasks, and the amount of amplitude decrease in response to global stimuli correlated with corresponding performance accuracy and response times (Johnson et al., 2005). Further evidence of N1 amplitude reduction (~200 post stimulus onset) is recently demonstrated in the fine-grain visual-masking discrimination task employed by (Plomp et al., 2013). Intriguingly, the extra time required by ScZ to reach normal discrimination performance levels did not alleviate the pronounced N1 amplitude reduction. Instead, as source analysis revealed, the discrimination difficulties are likely to be related to the significantly weaker parietal and lateral occipital activity in ScZ patients.

*N250*—a negative component that peaks ~250 ms over fronto-central electrode sites—is considered sensitive to the emotional content of faces (Streit et al., 1999, 2001). Some studies have found reduced N250 amplitude in ScZ patients (e.g., Streit et al., 2001; Wynn et al., 2008) with normal N170 suggesting emotional information decoding deficits, while others have found the opposite (e.g., Johnson et al., 2005; Turetsky et al., 2007) suggesting that facial feature encoding is impaired rather than emotional information decoding.

*N*_*CL*_—a negative component that manifests ~270–320 ms observed during visual tasks involving perceptual closure—is characterized by bilateral occipito-temporal scalp topography (Doniger et al., 2002) and is thought to reflect effortful extraction of object identity (Foxe et al., 2005). Significantly reduced *N*_*CL*_ amplitude has been shown to be preceded by a normal N1 but prominently reduced P1 amplitude in ScZ patients (Doniger et al., 2002; Foxe et al., 2005). This observation has led to a view that the initial stages of visual ventral stream processes are unaffected in ScZ patients but the later stages of ventral stream processing involving object recognition are likely affected by indirect magnocellular-mediated dorsal stream inputs (e.g., Merigan and Maunsell, 1993; Nowak and Bullier, 1997; Schroeder et al., 1998) into the visual areas along the ventral stream (e.g., lateral occipital cortex).

*P300*—a positive component that peaks ~300–900 ms post stimulus onset. Unlike earlier potentials, it is supposed to be an endogenous component which reflects stimulus context and levels of attention and arousal. The auditory P300 has consistently been shown to be impaired both in amplitude and latency (Bramon et al., 2004) while evidence for a dysfunctions during visual processing are less consistent (Ford, 1999); but see recent findings by Oribe et al. (2013) on prodromal and first-episode ScZ-patients.

Given that the visual system has been extensively explored through anatomy, electrophysiology and neuroimaging, detailed examination of visual dysfunctions in ScZ may allow insights into the underlying neurobiological correlates. In the following review, we will focus on the role of high-frequency neural oscillations because considerable evidence exists on the role of beta (13–25 Hz)/gamma (25–200 Hz) band activity in visual processing as well as their potential involvement in the pathophysiology of ScZ. We will first examine the role of high-frequency neural activity during normal visual perception emphasizing work from invasive and non-invasive electrophysiology followed by an overview of studies with EEG/MEG that have examined alterations in high-frequency oscillations in ScZ. In the final section, we will discuss potential mechanisms which could account for abnormal beta/gamma oscillations in ScZ as well as provide recommendation for future research.

## High-frequency oscillations and visual processing

### Invasive electrophysiology

The involvement of gamma-band oscillations in sensory processing was first described by Adrian and colleagues in the 1940s (Adrian, 1950). Local field potential recordings from the olfactory bulb of anesthetized cats, rabbits, and hedgehogs showed pronounced oscillations in the 40–60 Hz frequency range. Subsequently, Freeman and colleagues (Bressler and Freeman, 1980; Freeman and Skarda, 1985) reported correlations between 35 and 85 Hz activity and olfactory perception, suggesting that gamma-band oscillatory modulations are involved in information coding in the olfactory system (Freeman, 1991).

Crucial evidence for a mechanistic role of gamma-band activity in visual perception and cortical computations was obtained by Singer and colleagues in the late 1980s (Singer, 1999). Specifically, Gray et al. (1989) showed that action potentials generated by cortical cells are phase-locked to the oscillatory gamma rhythm and consequently neurons aligned their discharges with high temporal precision. In its original formulation, the “Binding by Synchrony hypothesis” (BBS; Singer, 1999) proposed that ensembles of neurons that preferentially respond to features of the same object should fire synchronously, whereas these same neurons should not synchronize their firing to features belonging to other objects or to the background. Over the years, this hypothesis has gained substantial attention (for critical reviews see Gray, 1999; Shadlen and Movshon, 1999; Singer, 1999; Uhlhaas et al., 2009).

There is, however, conflicting evidence for the BBS in the primate primary visual area (V1) with some studies failing to find evidence for a relationship between binding of stimulus features and synchronous gamma-band activity (e.g., Lima et al., 2010). Given the large number of visual areas in the primate brain (Van Essen and Gallant, 1994), it is conceivable that binding through oscillatory mechanisms occurs in higher visual areas. Candidate brain regions would be structures that have been shown to express strong gamma oscillations in response to visual stimulation, such as the middle temporal cortex (MT) and V4 areas (e.g., Kreiter and Singer, 1996; Fries et al., 2001; but see also Thiele and Stoner, 2003; Palanca and DeAngelis, 2005). Nonetheless, it is important to note that the temporal and spatial scales for binding might be smaller than previously assumed and therefore even V1 remains as a viable candidate for binding (Fries et al., 2007; Havenith et al., 2011; Nikolić et al., 2013). These observations highlight the need to employ more sophisticated analysis techniques for the detection of transient signals that may be important for BBS.

In addition to stimulus parameters (see Box 2), the amplitude, and frequency of high-frequency oscillations in visual cortices can also be influenced by cognitive variables, such as attention. Initial evidence was provided by Fries and colleagues (Fries et al., 2001) who showed that 35–90 Hz activity in macaque visual area V4 strongly increased when behaviorally relevant stimuli were within the focus of attention. More recently, the same group demonstrated that spatial attention can also result in a shift to higher gamma-band frequencies in V1 (Bosman et al., 2012). Similarly, Lima et al. (2010) demonstrated using plaid stimuli that selective attention to one of the directional components of the plaid pattern affected gamma-band power in a manner that resembled the power (and frequency) modulation when the actual contrast of the stimulus was increased. Additionally, V1 gamma spectral power in macaques was shown to increase with temporal expectancy for behaviorally relevant events.

Box 2 **Stimulus parameters and high-frequency neural oscillations**

High-frequency oscillations are modulated by several important parameters, such as color, contrast, presentation eccentricity, orientation, and speed. The complexity of natural images makes it difficult to systematically explore the influence of any particular features on brain activity. Thus, simplified stimuli that can be parametrically changed over a feature space are typically employed. In particular, gratings are commonly used because they produce strong responses in the gamma frequency range. A brief overview of these parameters and their observed influence on high frequency oscillations are listed below.

**Size**

The effect of grating size on the activity recorded from the macaque primary visual area (V1) is such that bigger gratings generate stronger oscillations, lower peak frequency, and decreased firing rates (Gieselmann and Thiele, 2008; Jia et al., 2013a). Human MEG studies replicated the positive relationship between stimulus size and gamma frequency power, but failed to reproduce the effect of size modulating peak gamma frequency (Perry et al., 2013).

**Position**

The frequency of gamma oscillatory response is dependent on the apparent eccentricity of the stimulus. Centrally presented gratings tend to generate higher frequencies than stimuli presented peripherally (Lima et al., 2010).

**Contrast**

Gratings with higher contrast are associated with higher firing rates and increased gamma peak-frequency (Ray and Maunsell, 2010). The strength of gamma-band oscillations initially increases with contrast but if the contrast is too high there is a tendency for the oscillations to reduce in power (Ray and Maunsell, 2010; Jia et al., 2013b; Roberts et al., 2013).

**Speed**

Gamma-band oscillations vary consistently with stimulus-velocity of bars (Gray et al., 1990) and gratings (Friedman-Hill et al., 2000; Lima et al., 2011). In humans, static gratings generate lower peak gamma frequencies than moving gratings (Swettenham et al., 2009; Muthukumaraswamy and Singh, 2013).

**Spatial frequency**

The spatial frequency of gratings is known to modulate gamma power (Adjamian et al., 2004) and firing rates (Lima et al., 2010) following an inverted U relationship. Human neuroimaging data suggest that the peak frequency of gamma response is tuned to a narrow band of spatial frequency (2–4 cycles per degree; cpd), peaking at 3cpd (Adjamian et al., 2004); a finding not currently observed in monkey neurophysiology (Lima et al., 2010).

**Orientation and direction**

Animal neurophysiology has shown that gamma power and frequency are tuned to stimulus orientation and the direction of stimulus motion (Feng et al., 2010; Jia et al., 2013b). However, these effects have not yet been demonstrated in humans.

**Noise levels**

Progressively adding noise over a high contrast grating reduces the amplitude of gamma-band oscillations and their peak frequency without altering the average firing rates (Jia et al., 2011, 2013b).

**Luminance profile**

The luminance profile of a grating also influences gamma responses, with square waves generating more gamma power than sinusoidal gratings (Muthukumaraswamy and Singh, 2008).

**Stimulus type**

Gratings formed by concentric circles have been shown to produce higher gamma power compared to regular gratings formed by straight parallel elements (Muthukumaraswamy and Singh, 2013).

**Stimulus complexity**

Increases in stimulus complexity may lead to dramatic reductions in gamma-band power and also to changes in peak frequency (Lima et al., 2010).

**Color**

Pure color isoluminant gratings have been shown to produce undetectable gamma oscillations in human MEG recordings, that otherwise manifested strong gamma responses to luminance contrast gratings (Adjamian et al., 2008).

### EEG-MEG studies

Following the initial findings in both anaesthetized and awake animals on the potential relationship with visual processing (Singer and Gray, 1995), high-frequency oscillatory responses to visual stimuli have also been documented in EEG/MEG and electrocorticographic (ECoG) in humans (Sauvé, 1999; Tallon-Baudry and Bertrand, 1999; Lachaux et al., 2005; Tallon-Baudry, 2009; Martinovic and Busch, 2011). Broadly three different categories of high-frequency responses can be distinguished (Box 3).

Box 3 **Measures of high-frequency oscillations**

Neural oscillations can be characterized by their frequency, amplitude, and phase. These characteristic features are derived through the time-frequency decomposition of electrophysiological signals by means of a Fourier, Wavelet or Multi-Taper analyses. These techniques estimate the strength (amplitude) and phase (time-variant angle) of the signal at a particular frequency range. A signal's phase consistency across trials, described as the phase-locking index, can be computed from its phase information (Lachaux et al., 1999, 2003). Apart from inter-trial oscillatory phase-locking, the same approach can also be used to assess the inter-areal synchrony of neural oscillations (Lachaux et al., 1999; Rodriguez et al., 1999; Gross et al., 2001; Varela et al., 2001; Siegel et al., 2008; Hipp et al., 2011). Importantly, this phase-locking index provides an estimation of neural synchrony irrespective of the amplitude of the oscillatory signal. This estimate of synchrony between signals is distinct from spectral coherence measures in which amplitude and phase information are intermixed (Gross et al., 2001).

Several different parameters of high-frequency activity can be distinguished which are frequently employed in the analysis of electrophysiological data (Tallon-Baudry et al., 1996; Roach and Mathalon, 2008).

1. Evoked oscillations are phase- and time-locked to the stimulus onset, occurring ~70–120 ms post stimulus onset. They are typically detected by averaging over a large number of single trials and then band-pass filtered at the frequency range of interest to assess the latency and peak amplitude of this averaged signal.
2. Induced oscillatory responses manifest later than evoked responses with latencies ~200–400 ms and are not phase- locked to stimulus onset. Estimates of phase and amplitude are derived via time-frequency transformations (e.g., Morlet wavelets or Short Time Fourier Transforms) at single-trial level prior to averaging across trials.
3. Repetitive presentation of a stimulus at a consistent frequency can lead to entrainment of neural activity at the stimulation frequency and is referred to as “steady-state” response (SSR, e.g., Herrmann, 2001). The peak of SSRs should typically correspond to the stimulation frequency, although responses to the harmonics of the stimulation frequency are also observed with reduced amplitude.

Evoked high frequency oscillatory responses are typically observed ~70–120 ms post stimulus with an occipital topography (e.g., Martinovic and Busch, 2011). Sources of evoked gamma activity during simple visual stimulus perception or object recognition have been localized to primary visual (Muthukumaraswamy et al., 2010), lateral occipital-temporal and inferior temporal cortical areas (Gruber et al., 2006). Amplitude and phase-locking of evoked high-frequency oscillations are modulated by stimulus properties. Corroborating invasive studies (Box 2), human neuroimaging research have also reported beta and gamma-band activity amplitude increases with contrast (Sannita et al., 1995; Schadow et al., 2007), stimulus duration, and size (Perry et al., 2013). In addition, spatial frequency modulates the power of high-frequency activity non-monotonically (Sannita et al., 1995; Tzelepi et al., 2000) and eccentricity decreases beta/gamma-band power (Busch et al., 2004; Fründ et al., 2007).

Due to their latency and topography, evoked high-frequency responses are likely to reflect feed forward driven responses (e.g., Butler and Javitt, 2005; Tobimatsu and Celesia, 2006; Martinovic and Busch, 2011). Early studies suggested that both amplitude and latency of evoked high-frequency activity were largely unaffected by experimental manipulations involving attention (e.g., Tallon et al., 1995; Tallon-Baudry et al., 1996, 1997). However, more recent findings (Herrmann et al., 1999; Frund et al., 2008) have challenged this view through demonstrating that top-down factors can impact on evoked gamma-band activity as well (e.g., Chaumon et al., 2009).

Following the link between binding of stimulus of elements into coherent representations and gamma-band oscillations in invasive recordings (Gray et al., 1989), several EEG and MEG-studies have also examined the role of gamma-band oscillations during perceptual organization (Lutzenberger et al., 1995; Revonsuo et al., 1997; Keil et al., 1999; Spencer et al., 2003; Grützner et al., 2010), demonstrating increased amplitude and synchrony of gamma-band activity during the construction of coherent object representations. More recently, intracranial EEG data have complemented this evidence (Lachaux et al., 2005).

While induced oscillations are strongly enhanced by top-down factors (Vidal et al., 2006; Melloni et al., 2007), several studies have indicated that basic stimulus parameters, such as orientation (Edden et al., 2009), spatial frequency (Hadjipapas et al., 2007; Perry et al., 2013), luminance (Adjamian et al., 2008), and motion (Swettenham et al., 2009) also influence the occurrence of high-frequency activity. These findings, thus, challenge a simple dichotomy between evoked and induced activity. Moreover, recent findings demonstrated that sub-bands of low (~30–60 Hz) and high (~70–120 Hz) gamma-band oscillations are flexibly recruited by both feed-forward and feedback processes. For example, 30–60 Hz activity showed increases with initial unconscious associative learning of target-specific context in a search-task while 70–120 Hz oscillations occurred regardless of stimulus contexts (Chaumon et al., 2009). Likewise, amplitude of low gamma-band activity increases with conscious visual awareness in contrast to attention-related gamma band activity at higher frequencies (Wyart and Tallon-Baudry, 2008). The close association between the modulation of both low and high gamma band activity and cognitive processes further suggests that different gamma band frequencies could support the dynamic formation of distinct assemblies that underlie specific behavioral or cognitive function through “multiplexing” neural signal transmission (Vidal et al., 2006; Wyart and Tallon-Baudry, 2008).

## Alterations in high-frequency neural oscillations during visual processing in schizophrenia

**Figure 1:**
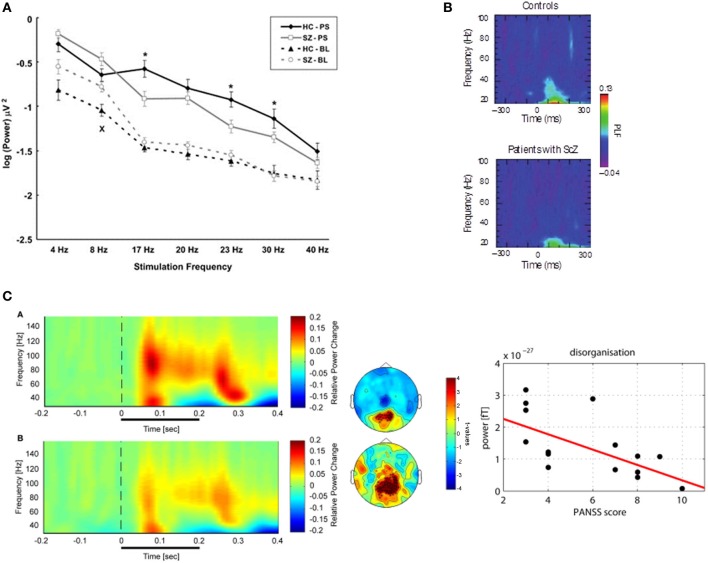
**(A)** Visual steady-state potentials (SSP) in patients with schizophrenia and controls: Average signal power for the two groups during resting state and photic stimulation at different frequencies recorded at Oz. (Legend: HC-PS, healthy control subjects during Photic Stimulation; SZ-PS, Schizophrenia subjects during Photic Stimulation; HC-BL, Healthy control subjects during Baseline; SZ-BL, Schizophrenia subjects during Baseline). Error bars indicate standard error. Significant differences between groups on ANOVAs are marked with “^*^” for the photic stimulation condition and “x” for the resting condition. Adapted from Krishnan et al. (2005). **(B)** Sensory evoked oscillations during a visual oddball task in patients with schizophrenia. The colored scale indicates the phase locking factor (PLF) of oscillations in the 20–100 Hz frequency range in the occipital cortex (electrode O1) for healthy controls and patients with schizophrenia. Control participants show an increase in phase locking for gamma oscillations ~100 ms after stimulus presentation. However, this is significantly smaller in patients with schizophrenia, indicating a dysfunction in early sensory processes. Adapted from Spencer et al. (2008). **(C)** High-Frequency Oscillations during Perceptual Organization in ScZ. Left-Panel: Time-frequency representations and topographies of gamma-band spectral power of MEG-data in response to Mooney faces for controls (top) and chronic ScZ patients (bottom). The gamma-band signal is expressed as relative power change in the post-stimulus time window compared to baseline, averaged across all channels. The topographies (middle panels) display the results for a non-Parametric ANOVA indicating the main effects of group for both low (top) and high (bottom) gamma-band oscillations at the sensor level. Intensity of red indexes increased activity in controls while stronger blue intensities suggest increased gamma-band power in schizophrenia patients relative to controls. The topographies depict corrected *t*-values and the channels that form a statistically significant cluster are indicated (^*^p < 0.001; ^*x*^*p* < 0.05). Right panel: Correlation between high gamma-band power and disorganization. The scatter-plot shows the relationship between high (60–120 Hz) gamma-band power in the 50–350 ms time window over positive channels and the disorganization component of the positive and negative syndrome scale. Adapted from Grützner et al. (2013).

The wealth of research highlights that high-frequency neural oscillations are involved in perceptual processing during normal brain functioning (Herrmann et al., 2004a; Tallon-Baudry, 2009; Martinovic and Busch, 2011). It is therefore conceivable that disturbances in the amplitude and synchrony of beta/gamma-band oscillations may have an important role in visual dysfunctions in ScZ. Indeed, a growing number of studies exploring this relationship have employed a range of visual tasks and assessed the integrity of the evoked and induced neural responses using different oscillatory parameters (see Table 1 for an overview and also Figure 1).

**Table 1:** **Summary of EEG/MEG studies investigating high-frequency neural oscillations in patients with chronic schizophrenia and healthy controls during visual perceptual tasks**.

**Paradigm**	**Imaging modality**	**Oscillatory measure**	**Parameter assessed**	**Main findings**	**References**
Steady state stimulation	EEG	Evoked	Amplitude	17–30 Hz range amplitude decrease over occipital electrodes	Krishnan et al., 2005
Backward masking	EEG	Evoked	Amplitude	30–40 Hz range amplitude decrease across electrodes	Wynn et al., 2005
	EEG	Evoked	Amplitude/Latency	30–35 Hz range amplitude decrease over parieto-occipital electrodes	Green et al., 2003
Oddball detection	EEG	Evoked	Inter-trial phase-locking	Decreased 30–38 Hz range phase-locking over parieto-occipital electrodes	Spencer et al., 2008
Illusory square	EEG	Evoked	Inter-trial phase-locking	Decreased 28–35 Hz range phase-locking over parieto-occipital electrodes	Spencer et al., 2004
		Induced	Inter-trial phase-locking	i) Decreased 30–45 Hz range phase-locking	
				ii) Decrease in peak phase-locking frequency (at 22–26 Hz cf. controls) over occipital and parietal electrodes in response-locked analysis	
		Evoked	Inter-trial phase-locking	i) Decreased 24–48 Hz phase-locking	
				ii) Decrease in peak phase-locking frequency in response to ‘No-Square’ stimuli over occipital and central electrodes	Spencer et al., 2003
		Induced	Inter-sensor phase-coherence	Long-range 20–26 Hz range decrease in phase-locking	
				Inter-hemispheric decrease in peak phase-coherence frequency (37–44 Hz cf. 48–57 Hz) particularly over posterior electrodes	
Mooney faces	MEG	Evoked	Amplitude	i) 25–140 Hz range amplitude decrease, especially pronounced in the 60–140 Hz range	Grützner et al., 2013
				ii) 25–60 Hz range fronto-central amplitude increase	
			Inter-trial phase-locking	Decreased 60–140 Hz range phase-locking	
		Induced	Amplitude	60–140 Hz range amplitude decrease over occipital sensors	
	EEG	Induced	Amplitude	Insignificant difference in the 40–70 Hz range across electrodes	Uhlhaas et al., 2006a
			Inter-trial phase-locking	Decreased and delayed onset latency of 20–55 Hz range phase-locking	
			Inter-sensor phase-coherence	Decreased 20–30 Hz phase-coherence between fronto-temporal and parieto-occipital electrodes	

Main findings of these studies are reported in brief, highlighting the frequency range of significant effects observed in patients cf. healthy controls.

### SSVEPs

Research investigating steady-state visually evoked potentials (SSVEPs) have observed reduced amplitude-modulation to repetitive stimulation at high but also at lower-frequencies in patients with schizophrenia relative to healthy controls. Krishnan et al. (2005) investigated SSVEPs to photic stimulation at frequencies from 4 to 40 Hz in EEG-recordings and reported decreased occipital amplitude modulation at 17, 23, and 30 Hz stimulation (Figure 1). In addition, higher “background noise,” which was defined as averaged power of neural activity 1 Hz above and below the photic stimulation frequency, was observed at frequencies 4–20 Hz in ScZ-patients. The data from SSVEPs parallel findings from auditory entrainment experiments suggesting a basic impairment of cortical circuits to support high-frequency activity in ScZ. In contrast to visual SSVEPs, however, auditory entrainment impairments have been predominantly demonstrated at 40 Hz frequency stimulation (Kwon et al., 1999). Although more recent data have also demonstrated entrainments deficits at 80 Hz as well as at theta-frequency ranges (Hamm et al., 2011).

### Evoked activity

Several studies have examined the integrity of evoked oscillations in ScZ using a variety of tasks. Backward masking paradigms are often used to assess early visual processing in ScZ. Given that basic features of any visual stimulus need to be integrated into a percept along the visual processing pathways, the effects of target percept masking could occur through the process of “integrating” the mask percept with the target percept, or through the process of “interrupting” the identification of target perception at a later stage of visual processing, or even via the process in which the target percept is “substituted” by that of the mask through a fast-acting process (Green et al., 2011). ScZ patients and unaffected siblings require longer inter-stimuli-intervals (ISI) between the target-mask stimuli for accurate feature identification of briefly presented targets (Green et al., 1994, 1997; Kéri et al., 2001). Depending on the type of masking (e.g., integration, interruption, or substitution; Green et al., 2011) the prolonged ISI interval has been linked to deficits in the magnocellular (M) and parvocellular (P) pathways (Schechter et al., 2003, 2005; Green et al., 2011).

The relationship between backward masking for location and object identification and gamma-band activity was examined in a series of studies by Green et al. (Green et al., 1999, 2003; Wynn et al., 2005). Systematic variation of inter-stimuli-intervals (ISIs) revealed that the response functions of ScZ-patients were best fitted with a continuous sine while in controls sensitivity to ISIs was consistent with a damped sinusoid (Green et al., 2003). Conversion of the wavelength parameter indicated that 30–35 Hz frequencies reflected best detection performance in controls. For ScZ-patients, the fitted sinusoids yielded a 32 Hz frequency conversion for the backward-masked location identification but a lower 15 Hz frequency for the backward-masked object identification.

To further link dysfunctions between backward masking and gamma-band activity, Green et al. (2003) also assessed EEG signals in response to backward masking of object identification in the 30–35 Hz frequency range. Peak latency in the 30–35 Hz spectral activity differed between groups with ScZ patients manifesting an earlier occipital-parietal peak around 100 ms while in controls gamma-bad activity was delayed (~200 ms), suggesting intact sensory registration in ScZ-patients. A follow-up study by Wynn et al. (2005) reported, however, reduced 30–40 Hz spectral power in ScZ patients between 50 and 200 ms during backward masking. In addition, while controls expressed stronger spectral activity to incorrect (vs. correct) trials, the opposite was observed for ScZ patients. It is presently unclear whether backward masking deficits involve impaired evoked oscillations or whether later processing stages might be compromised.

Deficits in high-frequency oscillations are also observed in response to basic sensory stimuli. Spencer et al. (2008) examined evoked EEG responses to auditory and visual stimuli in chronic ScZ patients (Figure 1). Interestingly, ScZ-patients' spectral amplitude and measure of inter-trial phase-locking to auditory stimuli were comparable to healthy controls. In contrast, the 25–45 Hz visually-evoked gamma oscillatory response was absent in the phase-locking frequency maps of ScZ patients, whose 30–38 Hz phase-locking over occipital regions was significantly reduced.

Similarly, ScZ-patients are characterized by reduced gamma-band responses to illusory square stimuli that presumably engage visual binding processes (Spencer et al., 2003, 2004). Spencer et al. (2003) showed that ScZ patients expressed a weaker P1 component which was accompanied by a reduced phase-locking of occipital evoked (24–48 Hz) activity to illusory square stimuli relative to controls. Moreover, ScZ patients' phase-locking over frontal-central EEG sensors were delayed in response to illusory squares, and occurred at lower frequencies. In a follow-up study, Spencer et al. (2004) examined response-time (RT) locked (20–45 Hz) evoked beta/gamma-band activity during the same paradigm and found reduced phase-locked activity in the 30–45 Hz frequency range in ScZ-patients which was accompanied by a shift to lower (22–26 Hz) activity relative to controls.

### Induced activity

Given that non-stimulus-locked (induced) oscillations, have been reported during perceptual organization processes during normal brain functioning (Rodriguez et al., 1999; Tallon-Baudry and Bertrand, 1999), it is likely that a focus on evoked activity only partially addresses the contribution of high-frequency activity toward visuo-perceptual dysfunctions in ScZ. To this end, two studies by Uhlhaas and colleagues (Uhlhaas et al., 2006a; Grützner et al., 2013) investigated induced beta/gamma spectral power during the viewing of Mooney faces, which involve the grouping of the fragmentary parts into coherent images based on the Gestalt principle of closure (Mooney and Ferguson, 1951). EEG-response to Mooney faces revealed largely intact gamma-band activity in ScZ-patients relative to controls (Uhlhaas et al., 2006a). However, a subsequent study with MEG (Grützner et al., 2013) reported prominent reduction in evoked and induced 60–120 Hz spectral activity in ScZ-patients (effect size: *d* = 1.26; Figure 1). Differences between the findings from EEG and MEG-data may be due to the fact MEG has improved sensitivity in detecting low-amplitude high-frequency oscillations than EEG (Muthukumaraswamy, 2013).

The findings of impaired induced gamma-band activity during perceptual organization are complemented by data showing reduced high-frequency activity during working memory and executive processes Haenschel et al. (2009) investigated gamma-band activity in EEG-data during a visual working memory paradigm demonstrating significant reductions in gamma-band power at higher working memory load conditions in early-onset ScZ-patients. Similarly, Cho et al. (2006) reported a decrease in induced gamma-band power in chronic ScZ-patients during a cognitive control task which involved the inhibition of a prepotent response.

### Long-range synchrony

In addition to the reduction in amplitude and consistency of evoked and induced spectral activity in ScZ patients, several studies have also assessed long-range neural synchrony through analyzing phase-synchronization between electrode pairs. This is of particular relevance because substantial evidence suggests that the functional networks underlying perception, attention, and executive processes rely on dynamic coordination through the inter-areal phase locking rhythmic activity (Lachaux et al., 1999; Varela et al., 2001). Spencer et al. (2003) observed a delayed onset of the 37–44 Hz phase synchrony as well as pronounced decreases in inter-hemispheric coherence during illusory-square perception over parietal electrodes in patients with ScZ. Moreover, Uhlhaas et al. (2006a) reported decreased phase-synchrony over fronto-temporal, and parieto-occipital sensors in the 200–300 ms period post stimulus onset, predominantly at beta (20–30 Hz) but also in the gamma-frequency range (31–38 Hz) during the perception of Mooney faces. The significant reductions in phase-synchrony observed in ScZ patients could indicate a global deficit in generating and sustaining synchrony both within local and also between distributed neural networks relevant for sensory processing.

### Relationships with clinical variables

Preliminary evidence suggests that alterations in high-frequency oscillations during visual processing in ScZ-patients may reflect psychopathological variables. Spencer et al. (Spencer et al., 2003, 2004) reported that evoked phase-synchrony during illusory square perception was correlated with conceptual disorganization and visual hallucinations as well as a relationship between the lowered oscillation frequency and the expression of positive symptoms (delusions) and conceptual disorganization. Finally, Uhlhaas et al. (2006a) reported a positive relationship between 40 and 70 Hz phase synchrony and positive symptoms while a reduction of phase-synchronization correlated with elevated negative symptoms. Significant correlations have also been reported with spectral power. Reduced 60–120 Hz spectral power was found to correlate with elevated levels of disorganization by Grützner et al. (2013). However, an important issue is whether these observed alterations in high-frequency activity are independent of medication status. To date, the only published finding by Minzenberg et al. (2010) indicated that gamma-band activity during cognitive control was reduced in medication-naïve FE-ScZ-patients.

## Pathophysiology of visual processing deficits and neural oscillations

Visually elicited high-frequency oscillations might be ideally suited for translations research (Spencer, 2009; Uhlhaas and Singer, 2012). In the following section, we review the potential involvement of changes in excitatory-inhibition balance, anatomical parameters, and genetic factors that could provide plausible explanations for the breakdown of high-frequency neural oscillations and the associated visual dysfunction observed in ScZ.

### Excitatory-inhibition (E/I) balance

One important parameter for the generation of high-frequency oscillations in visual circuits but also in the cortex in general is the balance between excitation and inhibition (E/I-balance). Convergence of theoretical (Spencer, 2009; Kopell et al., 2010) and empirical studies (Whittington et al., 1995; Wang and Buzsáki, 1996; Traub et al., 2004) indicate that the generation of high-frequency oscillations crucially involve networks of inhibitory interneurons (Whittington et al., 1995; Bartos et al., 2007; Mann and Paulsen, 2007; Buzsáki and Wang, 2012) and glutamatergically mediated excitatory drive (Lukatch et al., 2005; Chamberlain et al., 2012). Specifically, basket cells which express calcium-binding parvalbumim (PV; Cardin et al., 2009; Sohal et al., 2009; Volman et al., 2011) are of particular relevance for the generation of high-frequency oscillations, specifically at gamma-band frequencies, because of their fast-spiking properties (e.g., Buzsaki et al., 1983; Kawaguchi and Kubota, 1997).

More recently, optogenetic tools have enabled more precise links between changes in E/I-balance parameters and network oscillations to be established. For example, Sohal et al. (2009) showed that inhibition of PV interneurons led to an immediate suppression of 30–80 Hz oscillations while 10–30 Hz oscillations increased in power. In contrast, increasing PV-interneuron mediated feedback inhibition by boosting principal cell activity enhanced gamma-band power (Cardin et al., 2009).

Evidence suggests that E/I-balance parameters are disturbed in ScZ (Lewis et al., 2005, 2012). Specifically, the mRNA of GAD67 which synthesizes GABA is reduced in several cortical areas, including visual regions, in ScZ-patients (Akbarian et al., 1995; Mirnics et al., 2000; Hashimoto et al., 2003; Lewis et al., 2011, 2012). Moreover, this decrease is accompanied by reduced expression of the GABA membrane transporter 1 (GAT1; Volk et al., 2001; Lewis et al., 2005; Akbarian and Huang, 2006). GAT1 membrane transporters are expressed on chandelier neurons whose axon terminals synapse exclusively with the axonal initial segment of pyramidal neurons and thus uniquely regulate the excitatory pyramidal output (Lewis, 2000). Further evidence for a dysfunction in GABAergic transmission comes from magnetic resonance spectroscopy (1H-MRS) studies which have shown abnormal GABA-levels (Kegeles et al., 2012). Furthermore, MRS-measured reduction in GABA-levels was found to correlate with psychophysical impairment in orientation-specific surround suppression in ScZ patients (Yoon et al., 2010), suggesting a potential role in visual dysfunctions.

Additional parameters crucial for the generation of high-frequency oscillations include the AMPA- and NMDA-receptor-mediated activation of PV interneuron (Belforte et al., 2010; Carlén et al., 2012; Gonzalez-Burgos and Lewis, 2012). NMDA-receptor dysfunction has been implicated in the pathophysiology of ScZ through evidence from genetics (Carlén et al., 2012; Kirov et al., 2012) as well as from studies which have tested the impact of NMDA-receptor blockade on cortical processes. In healthy controls, Ketamine, an antagonist of the NMDA-receptor, elicits the full range of psychotic symptoms and impairments in cognitive processes, including visual perception (Hong et al., 2010). Furthermore, it has been shown in animal models that the blockade of NMDA-receptors induced aberrant high-frequency oscillations in extended cortical and subcortical networks (e.g., Hunt et al., 2011; Kittelberger et al., 2012; Phillips et al., 2012). For example, Anver et al. (2011) showed that NMDA-antagonists reduced the frequency of gamma-band oscillations as well as induced phase coupling of the normally independent generating networks in cortical layers III and V. These findings suggest that E/I-balance is crucial in assuring coordinated occurrence of high-frequency activity during normal brain functioning in networks involved in visual processing. Consequently, abnormalities in these parameters could lead to changes in both amplitude and synchrony of beta/gamma-band oscillations and, in turn, lead to visual deficits.

### Anatomical parameters

In addition to the crucial contribution of GABAergic and glutamatergic neurotransmission towards high-frequency oscillations, anatomical parameters such as the layout of excitatory long-range connections have been implicated in long-range synchronization and the integrity of visual processing (Engel et al., 1991). Synchronization of oscillatory activity in the beta and gamma frequency range is dependent on cortico-cortical connections that reciprocally link cells situated in the same cortical area, in different areas, or even in different hemispheres (Engel et al., 1991). Interestingly, a recent study demonstrated that callosal connections contribute to the subjective experience of a visual motion stimulus that requires inter-hemispheric integration (Genc et al., 2011). As such, disruptions in the volume and organization of anatomical connectivity could impair long-range synchronization and impact on visual processes that require large-scale integration. However, a recent study that investigated inter-hemispheric transfer times with ERPs did not support this hypothesis in ScZ (Whitford et al., 2011a).

Further research is required to examine, in greater detail, the relationship between anatomical abnormalities and high-frequency oscillations. This is particularly relevant given the evidence from *in vivo* and post-mortem studies in patients with ScZ highlighting that both the volume and organization of white matter is abnormal, including both early and higher visual areas (Akbarian and Huang, 2006; Hashimoto et al., 2008). Additional evidence supporting the abnormal anatomy of visual regions was reported by Selemon et al. (1995) who observed increased neuronal density in area 17 (occipital cortex) as well as in area 9 (frontal cortex) in ScZ-patients. In contrast, Dorph-Petersen et al. (2007) found no difference in neuronal density in area 17 in ScZ and cortical thickness was in the normal range. However, the authors reported significantly reduced number of neurons as well as volumetric decreases in area 17 (Dorph-Petersen et al., 2007).

Abnormalities in gray matter could also potentially impact on the amplitude of neural oscillations as EEG/MEG signals are dependent on the ionic currents flowing in the dendrites of clusters of synchronously activated neurons during synaptic transmission that could be compromised by either reductions in the number of neurons and/or decreases in neuropil. Supporting a potential link between electrophysiological parameters and anatomical abnormalities in ScZ, Onitsuka et al. (2006) demonstrated an association between the degree of gray matter reduction and a decrease in the N170 ERP component.

### Genes

With the genetic heritability of ScZ being estimated to be around 50–80% (e.g., Sullivan et al., 2003; Harrison and Weinberger, 2004), one plausible hypothesis is that ScZ risk genes may influence the strength and frequency of neural oscillations in the disorder. Indeed, recent animal models which have examined the effects of risk genes on changes in high-frequency oscillations support this view. Carlson et al. (2011) investigated gamma-band responses during auditory stimulation in mice with reduced dysbindin-1 expression which is a major ScZ risk gene (Straub et al., 2002; Ross et al., 2006; O'Tuathaigh et al., 2007). Deficits in early evoked gamma-band activity were found which were associated with a decrease in PV cell immune-reactivity. Similarly, Fisahn et al. (2009) demonstrated that polymorphisms in genes encoding Neuregulin-1 (NRG-1) and one of its receptors (ErbB4) selectively increase the power of *in vitro* gamma-band oscillations in hippocampal slices. Accordingly, these data suggest the possibility that ScZ-risk genes modify the E/I-balance parameters which in turn dysregulate the occurrence of high-frequency oscillations.

**Figure 2:**
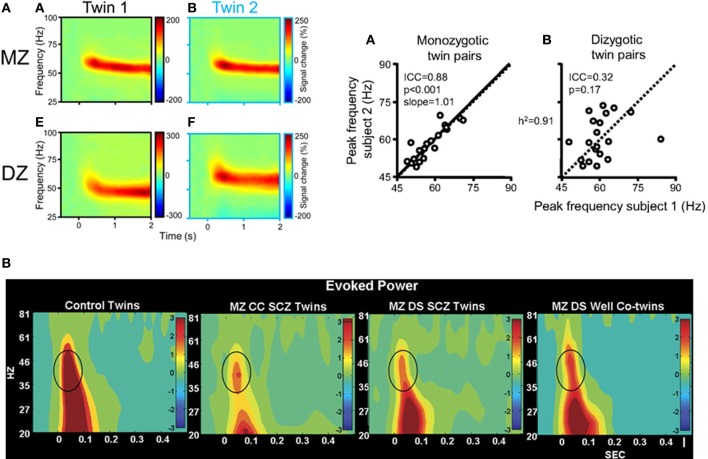
**(A)** Left: visually induced gamma-band activity in MEG data in a monozygotic (MZ) and a dyzygotic (DZ) twin pair. Time-frequency representations (TFRs) of activity in the gamma-band range relative to prestimulus baseline levels in two twins of a MZ pair, averaged across 74 parieto-occipital MEG sensors. Time 0s denotes stimulus onset. Right: correlation between gamma-peak frequencies in MZ twins [A] and DZ twins [B]. Each data point represents the peak frequency of one twin vs. that of his or her co-twin (random axis assignment). Slope values are estimated by random permutations of *x* and *y* values. The data suggest a heritability of the gamma-band frequency of 91%. Adapted from van Pelt et al. (2012). **(B)** Evoked oscillatory activity in schizophrenia patients and their unaffected co-twins. EEG time-frequency analyses of evoked gamma-band power during an auditory oddball task for responses to the standard stimuli at electrode Cz in healthy twins, MZ twins concordant with schizophrenia, MZ twins discordant with schizophrenia, and unaffected co-twin members. Impaired evoked gamma-band power was significantly associated with schizophrenia and unaffected co-twins exhibited significantly reduced 30–60 Hz power as well-compared with controls, highlighting the genetic contribution toward impairments in high-frequency oscillations in the disorder. Adapted from Hall et al. (2011) by permission of Oxford University Press.

Recent evidence from genetic studies which have investigated the heritability of visually-induced gamma-band oscillations supports the utility of using high-frequency activity as an intermediate phenotype. van Pelt et al. (2012) recorded visually induced MEG-activity in monozygotic and dizygotic twin pairs (Figure 2). The peak-frequency of gamma-band oscillations were highly correlated in monozygotic but not in dizygotic twins, highlighting a strong genetic determination of gamma-band oscillations. Additionally, reduced auditory evoked gamma-band activity has been demonstrated in first-degree relatives of patients with schizophrenia as well as in unaffected, monozygotic twins with a high degree of heritability (Hall et al., 2011; Figure 2). Moreover, Hong et al. (2008) showed that abnormalities in theta- and alpha-band oscillations during sensory gating in ScZ-patients, their relatives, and healthy controls were characterized by heritability rates that exceeded those of traditional ERP measures.

## Issues for future research

The current review has shown a close relationship between visual processing and high-frequency oscillations during normal brain functioning as well as a potential link between aberrant beta/gamma-band activity and dysfunctional visual perception in ScZ. Given the known neurobiological parameters involved in the generation of high-frequency oscillations, we suggest that visually elicited high-frequency oscillations may constitute a useful window for gaining further insights into the pathophysiology of ScZ. To this end, we would like to raise several issues that we consider critical for future research.

The overall conclusion that can be drawn from the studies reviewed is that ScZ is associated with reductions in the amplitude, frequency, and/or synchronization of beta/gamma-band oscillations during visual processing. Such deficits have been demonstrated during a wide range of task-conditions, such as in basic responses to visual entrainment (e.g., Krishnan et al., 2005), impaired stimulus-locking of oscillatory activity during perceptual binding (Spencer et al., 2003, 2004, 2008) and visual masking (Green et al., 1999, 2003; Wynn et al., 2005) as well as deficits in generating high-frequency oscillations (Grützner et al., 2013) and their large-scale integration during perceptual organization of complex stimuli (Spencer et al., 2003; Uhlhaas et al., 2006a,b). Abnormalities in visually elicited high-frequency oscillations are consistent with reduced beta/gamma-band activity during auditory (Kwon et al., 1999) and somatosensory perception (Arnfred et al., 2011). Together these findings suggest that cortical circuits in ScZ may be characterized by a comprehensive impairment in the mechanisms responsible for the generation and coordination of adequate high-frequency activity that is present in multiple regions and networks.

### High-frequency oscillations, the visual system and ScZ

Psychophysical evidence has shown that ScZ-patients are characterized by several deficits in visual processing which include a deficit in stimuli involving the magnocellular pathway (Butler et al., 2008; Javitt, 2009), reduced contextual integration (Yoon et al., 2010; Dias et al., 2011; Yang et al., 2013) and dysfunctions in perceptual organization (Uhlhaas and Silverstein, 2005). Given that the amplitude and the frequency of beta/gamma-band oscillations are closely related to stimulus properties during normal brain functioning (see Box 2), a combination of precise manipulation of stimulus parameters and electrophysiological approaches may yield novel insights into the relationship between visuo-perceptual dysfunctions and high-frequency oscillations in ScZ.

A reported core deficit underlying visual dysfunction in ScZ is the gain control of visual neuronal responses in ScZ (Butler et al., 2008). Gain control refers to the ability of neurons to modulate their response amplitude and constitutes a general feature of cortical computations (Salinas and Thier, 2000). Impairments in gain control in ScZ are supported by reduced contrast sensitivity (Yang et al., 2013), impaired motion perception (Kim et al., 2006; Chen, 2011) and contextual effects (Tadin et al., 2006; Yang et al., 2013; see also Butler et al., 2008 for a review). Moreover, neurophysiological studies have provided psychophysical evidence that these stimulus parameters, which are differentially processed in ScZ, modulate high-frequency activity. For example, increasing the contrast of visual stimuli enhances the frequency of the gamma-band rhythm in V1 (Ray and Maunsell, 2010) and V2 (Roberts et al., 2013). Similar findings have been observed for motion whereby static gratings are associated with lower peak frequencies than moving gratings (Gray et al., 1990; Swettenham et al., 2009; Muthukumaraswamy and Singh, 2013; see Box 2). Given these robust relationships, one option for future studies is to parametrically manipulate stimulus contrast and velocity and assess changes in high-frequency oscillations which could yield insights into the integrity of visual circuits in ScZ to support the occurrence and amplitude tuning of beta/gamma-band oscillations.

While such experiments are potentially important for probing dysfunctions in early visual regions, oscillatory dynamics are also crucially involved in mediating the influences of neuronal activity generated in anterior brain regions over the early stages of visual processing, such as during attention (Womelsdorf and Fries, 2007). Of relevance, evidence supporting the facilitatory effects of attention processes, particularly spatial attention, on visually induced high-frequency oscillations constitutes an additional example of gain control whereby neuronal responses to stimuli at attended locations are increased relatively to non-attended locations (Hillyard et al., 1998). This gain in neural response could be mediated through changes in the synchrony of inhibitory networks (Tiesinga et al., 2004a,b).

Previous psychophysical research in ScZ has implicated deficits in the utilization of top-down mediated cues (Silverstein et al., 1996) as well as dysfunctions in bottom-up driven processing in early visual pathways (Uhlhaas and Silverstein, 2005; Butler et al., 2007), suggesting that effects of attention on neural oscillations could be relevant in order to disentangle the contribution of feed-forward mediated vs. top-down processes toward visual deficits in ScZ. Recent studies from invasive electrophysiology (Fries et al., 2001; Ray et al., 2013) as well as from EEG/MEG (Wyart and Tallon-Baudry, 2008) have demonstrated that attention can lead to an increase in both the amplitude as well as the frequency shift of gamma-band activity (Kahlbrock et al., 2012; Koelewijn et al., 2013). These effects occur in early visual areas (Koelewijn et al., 2013) as well as at higher brain areas (Tallon-Baudry et al., 2005) and are accompanied by changes in the coherence of oscillations between early and higher visual regions (Siegel et al., 2008; Bosman et al., 2012). Therefore, detailed probing of attention effects on high-frequency oscillations could potentially offer insights into the differential (e.g., anatomical and frequency-specific) contribution of both bottom-up and top-down processes toward visual processing abnormalities in ScZ.

### Visual perception, high-frequency oscillations in ScZ and translational research

One important issue concerns the possibility of distinct roles of beta and gamma-band oscillations during visual processing. Recent research have highlighted that beta-band oscillations mediate mainly top down activity and hence are critically involved in the prediction of upcoming sensory events while gamma-band oscillations, at least in sensory cortices, are involved in feed-forward signalling (Buschman and Miller, 2007; Arnal and Giraud, 2012). This distinction is supported by the differential laminar expression of beta and gamma-band oscillations. *In vitro* and *in vivo* recordings show that gamma-band activity is prominently generated in superficial layers 2/3 of the cortex (Buffalo et al., 2011), the main origin of feed- forward connections, and dependent upon fast, transient excitation of fast-spiking interneurons via metabotropic glutamate receptors (Whittington et al., 1995). In contrast, beta oscillations are mainly found in infragranular layers, from which feed-back projections originate preferentially. Interestingly, the generation of beta-band oscillations can be independent from excitatory or inhibitory synaptic transmission (Roopun et al., 2008). These observations provide potential hypotheses for future studies to investigate the differential contribution of beta/gamma-band oscillations during visual processing in ScZ. In particular, these investigations could be combined with the investigation of attention effects to address the potentially distinct roles of feed-forward vs. top-down mediated neuronal activity in perceptual dysfunctions in the disorder.

In addition to the modulation of beta/gamma-band power and synchrony, changes in the oscillatory peak-frequency may also be useful in establishing links between non-invasive EEG/MEG-measures and E/I-balance parameters (see Spencer et al., 2004; Ferrarelli et al., 2012). It is conceivable that the frequency at which a network oscillates may more closely mirror biophysical parameters of the underlying network. For example, the deactivation kinetics of different GABAergic receptors strongly impact on the generation of fast vs. slow GABAergic currents which in turn are an important parameters for the frequency of oscillations (Wang and Buzsáki, 1996). Additionally, the peak-frequency of visually induced gamma-band activity in MEG-data has been shown to be under close genetic control (van Pelt et al., 2012), indicating that the frequency of gamma-band oscillations could be linked to genetically determined differences in channel-subunits.

Furthermore, mechanistic links between disturbed oscillations and visual perception in ScZ may also be established in combination with rhythmic stimulation through transcranial magnetic stimulation (TMS) and transcranial alternating current stimulation (tACS). Available evidence suggests that oscillatory brain processes can be entrained, enhanced or perturbed by means of external stimulation (Romei et al., 2011; Thut et al., 2011a,b; Antal and Paulus, 2013), which raises the possibility of targeting specific oscillations frequencies in conjunction with visuo-perceptual processes in patients ScZ. The feasibility of using TMS, for example, to probe neural circuits in ScZ has been demonstrated in several recent studies (Ferrarelli et al., 2012; Frantseva et al., 2012).

Finally, future research should also consider the overlap in visually elicited high-frequency dysfunctions with related disorders, such as bipolar (BP) and autism spectrum disorders (ASDs). There is substantial evidence that ASDs are characterized by impairments in visual processing as well as deficits in high-frequency oscillations (Dakin and Frith, 2005; Sun et al., 2012). Similarly, there is evidence for impairments in bipolar disorder because auditory-steady state responses (O'Donnell et al., 2004) as well as long-range coherence (Özerdem et al., 2010) at gamma-band frequencies are significantly impaired.

Given the substantial overlap in genes, cognitive deficits and clinical symptoms between different diagnostic categories, it also conceivable that neural oscillations can be used to assign patients into novel categories based on neural oscillations. Fingerprints of neuronal dynamics, such as alterations in the frequency, temporal precision, phase locking, and topology of neuronal oscillations, during visual processing provide a rich coding-space for the definition of discrete entities or taxon (Meehl, 1992) within and also between diagnostic categories. As such, the close links between genes, neurobiology, and parameters (Figure 2) are perhaps well-suited to identify pathways mediated by risk genes.

### Beta/gamma-band oscillations and low-frequency activity

While the current review focused on activity at beta/gamma-band frequencies, activity in lower frequencies ranges (e.g., delta, theta, alpha bands) may also be potential targets for understanding visual dysfunctions in ScZ. Existing evidence from EEG-studies suggests impaired amplitude and phase-locking during visual stimulation is not confined to beta/gamma-band frequencies (Haenschel et al., 2010; Hamm et al., 2012).

The alpha-band rhythm (8–12 Hz) is particularly relevant for the understanding of visual perception as the alpha cycle modulates perceptual detection rates (Valera et al., 1981; Dugué et al., 2011). Moreover, there is consistent evidence that oscillations in the alpha-band interact with the amplitude of gamma activity through cross-frequency coupling (Osipova et al., 2008), raising the possibility that impairments in high-frequency activity could also result from an impaired hierarchical organization of oscillations.

In addition to cross-frequency interactions, there is growing consensus that lower-frequency rhythms also play an important role in coordinating sensory predictions within and between modalities (see Schroeder and Lakatos, 2009 for a review). Recent work by Lakatos et al. (2013) demonstrated that impaired sensory discrimination of auditory stimuli in ScZ-patients was correlated with a deficit in effectively entraining inter-trial delta phase-locking to anticipate relevant sensory processing, and a failure to suppress task-irrelevant activity. These findings highlight the potential relevance of sensory predictions for auditory processing impairments in ScZ. It remains to be investigated whether predictive mechanisms in the visual domain are similarly affected in ScZ.

### Methodological implications

While it is possible that alterations in high-frequency oscillations during visual processing may reflect dysfunctions in specific variables involved in the generation of high-frequency activity, we cannot exclude the possibility that several non-specific factors, such as the impact of antipsychotic medication, chronic stress and the non-neuronal origin of certain EEG/MEG-signal components, contribute toward findings of impaired beta/gamma-band oscillations in ScZ patients. Accordingly, advances in analytic techniques and experimental designs are essential in order to allow clearer links between changes in high-frequencies oscillations and visuo-perceptual deficits in ScZ.

An approach to further identify such relationships is to employ single-trial analysis of EEG/MEG-data in combination with variation of stimulus parameters. At present, EEG/MEG-studies investigating high-frequency oscillations in ScZ have predominantly concentrated on differences in amplitude and peak-frequency values calculated across conditions or groups of participants. Given the substantial variability in behavioral and electrophysiological parameters both within and between groups, analysis of single-trial EEG/MEG-data analyses could potentially yield additional information as it allows a systematic mapping between brain activity and stimulus information as well as with indexes of behavioral variability (Pernet et al., 2011).

Furthermore, high-frequency oscillations during visual stimulation are accompanied by several important sources of artifacts which can resemble neuronally generated gamma band oscillations, and thus make the interpretation of EEG/MEG-signals difficult. Specifically, induced gamma-band activity coincides with the maximal frequency of micro-saccades which elicit a saccadic spike potential (SSP). Seminal work by (Yuval-Greenberg et al., 2008) highlighted that the SSP can mimic gamma oscillations in bandpass-filtered EEG signals if artifact-correction procedures are not adequately employed (Melloni et al., 2009). The presence of SSP-related gamma-band activity has also been recently demonstrated in MEG-recordings (Carl et al., 2012). Additionally, muscle artifacts can constitute another non-neuronal source of high-frequency activity that, if not carefully removed, can simulate power modulations in the gamma band range over visual regions (Whitham et al., 2007; see also Hipp and Siegel, 2013; Muthukumaraswamy, 2013 for recent reviews of these issues).

In addition to the contribution of eye-movement related artifacts toward high-frequency signals, a potentially important issue is also the relationship between neural oscillations, eye-movements, and visual dysfunctions. Abnormalities in the several eye-movement parameters such as smooth pursuit, fixation stability, scan-path, and fixation dispersal during free viewing are one of the more robust domains of impairments in ScZ-patients (e.g., Benson et al., 2012). Moreover, recent findings suggest that patterns of saccades strongly modulate the occurrence of high-frequency oscillations in V1 (Bosman et al., 2009) and that different oscillatory frequencies are involved in the organization of eye-movement patterns (Ito et al., 2011). Indeed, disentangling the relationship between high-frequency oscillations, eye-movements, and visual processing dysfunctions will be an important albeit challenging area of research.

## Summary

The findings reviewed suggest a potential link between the occurrence of beta/gamma oscillations and the pronounced deficits in visual perception in ScZ. Evidence supporting such a relationship comes from EEG/MEG studies indicating reductions in synchrony and amplitude of beta/gamma-band oscillations during basic and complex visual stimuli as well through anatomical findings that highlight impaired structure and composition of visual circuits in the disorder. Importantly, given the known mechanisms involved in the genesis of high-frequency oscillations, the evidence and clinical importance of visual dysfunctions in ScZ, as well as the opportunity to measure high-frequency oscillations non-invasively, visually elicited high-frequency oscillations in ScZ are potentially suited for translational research.

### Conflict of interest statement

The authors declare that the research was conducted in the absence of any commercial or financial relationships that could be construed as a potential conflict of interest.
